# Ionomic Approaches for Discovery of Novel Stress-Resilient Genes in Plants

**DOI:** 10.3390/ijms22137182

**Published:** 2021-07-02

**Authors:** Sajad Ali, Anshika Tyagi, Hanhong Bae

**Affiliations:** 1 Department of Biotechnology, Yeungnam University, Gyeongsan 38541, Korea; sajadali84@gmail.com; 2 National Institute for Plant Biotechnology, New Delhi 110012, India; tyagi.anshika9@gmail.com

**Keywords:** ionomics, biotic stress, abiotic stress, gene identification, QTL mapping, omics, elemental analysis

## Abstract

Plants, being sessile, face an array of biotic and abiotic stresses in their lifespan that endanger their survival. Hence, optimized uptake of mineral nutrients creates potential new routes for enhancing plant health and stress resilience. Recently, minerals (both essential and non-essential) have been identified as key players in plant stress biology, owing to their multifaceted functions. However, a realistic understanding of the relationship between different ions and stresses is lacking. In this context, ionomics will provide new platforms for not only understanding the function of the plant ionome during stresses but also identifying the genes and regulatory pathways related to mineral accumulation, transportation, and involvement in different molecular mechanisms under normal or stress conditions. This article provides a general overview of ionomics and the integration of high-throughput ionomic approaches with other “omics” tools. Integrated omics analysis is highly suitable for identification of the genes for various traits that confer biotic and abiotic stress tolerance. Moreover, ionomics advances being used to identify loci using qualitative trait loci and genome-wide association analysis of element uptake and transport within plant tissues, as well as genetic variation within species, are discussed. Furthermore, recent developments in ionomics for the discovery of stress-tolerant genes in plants have also been addressed; these can be used to produce more robust crops with a high nutritional value for sustainable agriculture.

## 1. Introduction

In modern agriculture, abiotic and biotic stresses are the leading causes of reduced crop yield and productivity, resulting in significant economic losses [1]. In the future, global warming will intensify the impact of these stresses on sustainable agriculture and pose a serious threat to food security [2,3]. Additionally, it will also lead to a mounting concurrence of biotic and abiotic stress combinations in crops, which would be detrimental to their growth and productivity [4]. On the other hand, nutritional security is becoming one of the major challenges for researchers developing not only stress-resilient crops but also biofortified crops to tackle malnutrition. Therefore, a deep understanding of crop responses to multiple stressors is important for improving crop resilience in sustainable agriculture [5]. Plants undergo a series of morphological, biochemical, physiological, and molecular modifications in order to combat the effects of environmental stresses [6]. The functioning of these stress-resilient pathways in plants is primarily dependent on the availability and accumulation of minerals. Recently, ions (both essential and non-essential) have been identified as key players in plant stress biology due to their multifaceted functions, thereby creating new areas of investigation [7]. In this regard, ionomics, a multidisciplinary field, can provide new crop platforms for identifying genes and their regulatory pathways related to mineral accumulation, transportation, cross-talk, and stress resilience. Furthermore, such platforms will enable the development of ionomics-based biomarkers that can assess whether a plant has attained a certain biochemical or physiological state under different stresses and environmental conditions. For example, Zn deficiency-responsive genes/biomarkers like *ZIP4* and *IRT3* have been reported in Arabidopsis that will be useful proxies for measuring zinc deficiency tolerance [8]. Plants need many inorganic mineral elements to complete their life cycle, as well as to maintain healthy growth. Minerals play a vital role in various biological functions as cofactors, macromolecules, osmotic solutes, and ionizing species [9]. Generally, minerals are classified as macronutrients and micronutrients, or essential elements based on the quantity required by plants. Macronutrients, namely, potassium (K), nitrogen (N), phosphorus (P), calcium (Ca^2+^), magnesium (Mg), and sulfur (S), are required by plants in higher quantities (>0.1% of dry weight). On the other hand, micronutrients such as zinc (Zn), manganese (Mn), iron (Fe), chlorine (Cl), copper (Cu), boron (B), molybdenum (Mo), and nickel (Ni) are required in smaller quantities (<0.1% of dry weight) [10]. In addition to these essential elements, plants take up many non-essential elements such as aluminum (Al), silicon (Si), selenium (Se), sodium (Na^+^), cobalt (Co), gallium (Ga), cadmium (Cd), and arsenic (As) [11]. At a specific uptake level, some of these non-essential elements are beneficial for certain plant species. For example, Si, which was recently declared a useful element for plant growth by the International Plant Nutrition Institute (IPNI), confers incredible benefits onto plants, particularly under stress conditions (http://www.ipni.net/nutrifacts-northamerican, accessed on 1 April 2021). Elements such as Cd and As have no biological function and are not required by plants, but are often taken up, and cause severe toxicity [12]. However, plants have developed a variety of protective mechanisms to cope up heavy metal stresses, which includes decreased heavy metal absorption, sequestration of metal into vacuoles, binding phytochelatins/metallothioneins, and activation of various antioxidants [13]. Crops are the principal sources of essential mineral nutrients for humans and animals. Therefore, understanding their elemental composition is essential for healthy crop production.

Ionomics is the study of metal, non-metal, and metalloid compositions in living organisms using high-throughput elemental analysis technologies such as inductively coupled plasma-atomic emission spectroscopy (ICP-AES) and ICP-mass spectrometry (ICP-MS) [14]. In a broader sense, it is the study of an organism’s ionome and is aimed at quantifying as many elements as possible in individual samples and at determining how metabolism, genetics, development, and the environment influence the elemental composition of target organs, tissues, and cell types [15]. The concept of the ionome was introduced about 10 years ago, and since then, significant progress has been made in the area of ionomics, mainly because of its integration with genetics and other high-throughput tools to identify the genes that regulate the ionome [16,17]. The initial work on ionomics in the model plant *Arabidopsis thaliana* has become the key for its expansion to agriculturally relevant crops [16]. Ionomics has been used to investigate the role of minerals in different economically important crops, namely, *Glycine max* [18], *Zea mays* [19], *Oryza sativa* [20], *Brassica napus* [21], *Olea europaea* [22], and *Vitis vinifera* [23]. It has wide applications for studies on nutrient utilization, environmental monitoring, biofortification, and food safety [15]. In addition, this high-throughput tool is efficient for revealing not only interdependency among various mineral elements but also the genetic factors that regulate mineral transport and homeostasis in plants [24,25]. Moreover, it may also be used to investigate the effects of phylogenetics and the climate on plant mineral accumulation [26]. Ionomics has been applied extensively in plant biology, not just for human food requirements but also to study plant developmental and stress biology. Shakoor et al. [27] revealed how ionomics could tackle malnutrition and improve food safety. They performed ionomic analyses of staple food crops such as cereals and beans. Ionomics, when used in conjunction with other high-throughput tools, such as proteomics, transcriptomics, and metabolomics, has the ability to bridge the gap between the understanding of a genotype and the phenotype it regulates [14]. In ionomics, mutant screening and natural variation are useful to identify genes and alleles that are essential for elemental accumulation and variations in the ionomes of different genotypes [28,29]. The most comprehensive data on ionomics applications have been generated using *A. thaliana* through a mega project at the Purdue Ionomics facility (http://www.ionomicshub.org, accessed on 1 April 2021). This venture has played an outstanding role in the identification of numerous ionome regulatory genes [30]. There are total 1764 genes from *A. thaliana* (136), *O. sativa* (141), *Medicago truncatula* (176), *Triticum aestivum* (267), *Z. mays* (152), *G. max* (268), *Populus trichocarpa* (197), *Sorghum bicolor* (135), *Setaria italica* (146), and *Setari viridis* (146), which have been identified as ionome regulatory genes. Some of the primary genes and their elemental targets have been shown in Table 1. The Purdue Ionomics Information Management System (PiiMS) has been used to record all data generated from such projects (http://www.ionomicshub.Org, accessed on 1 April 2021). In plant stress biology, ionomics-based biomarkers can help determine whether a plant has attained a specific biochemical or physiological state under various unfavorable environmental conditions. Subsequently, they can also help screen plants that are more prone to abiotic and biotic stresses; this is not possible using other high-throughput tools. In addition, ionomics has revealed genes that govern natural variation in the plant ionome and play a crucial role in stress resilience. In this review, we focus on the role of ionomics in crop improvement, particularly in improving abiotic and biotic stress tolerance.

## 2. Importance of Ionomics in Human Health, Food, and Agriculture

**Figure 1:**
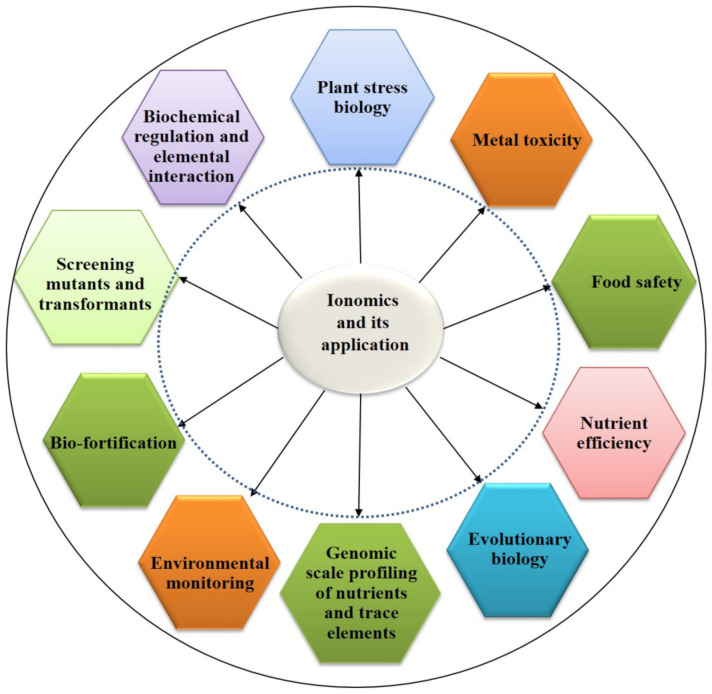
Overview of application of ionomics in human health and agriculture to identify different ions under diverse environmental conditions.

Mineral nutrition is extremely important for the proper functioning of growth and developmental processes in living organisms. Seventeen elements are essential to plants; however, plants also take up and accumulate non-essential elements [11]. On the other hand, humans need at least 25 elements [70], which they mainly get from plants. In developing and developed countries, one of the widespread issues related to public health is mineral malnutrition; approximately two-thirds of the global population is at risk owing to a deficiency in one or more basic mineral elements [70]. One of the principal causes of malnutrition in developing countries is Fe and Zn deficiency [71]. Biofortification, as a strategy, could potentially address malnutrition through development of crops containing vital mineral elements and by enhancing their nutritional importance for humans [72]. However, a complete understanding of the mechanism of mineral nutrient accumulation in grains is required before this technology can attain its full potential. In this context, high-throughput elemental profiling plays an important role in determining the capacity of mineral absorption genes in model and crop plants. Many mineral rich biofortified crops such as iron beans, iron pearl millet, zinc wheat, zinc rice, zinc maize, were successfully produced, tested, released, and marketed by HarvestPlus partnering institutions to farmers and consumers. The potential benefit of these biofortified crops has received widespread recognition for its ability to reduce the risk of Zn and Fe deficiency in underdeveloped nations [73]. Recently, Iron for Adolescent (FeFA) Girls, a new initiative to address malnutrition among adolescents, was recently launched by ICMR-National Institute of Nutrition (NIN) in association with International Crops Research Institute for the Semi-Arid Tropics (ICRISAT) for the development of Fe biomarkers using various omics approaches, including ionomics, to overcome iron deficiency known as anemia (https://www.icrisat.org/tag/fefa-girls/, accessed on 20 June 2021). Furthermore, only a few biofortified vegetable products are available on the market, such as selenium-enriched potatoes, carrots, and onions (Selenella), and iodine-biofortified potatoes ‘Iodì’ [74]. In contrast, certain crops pose a threat to public health, as they contain some toxic elements. The edible parts of crops contain a high concentration of toxic elements when plants are grown on cultivable land polluted with heavy metals and metalloids. In some Asian countries, many studies have been undertaken to address the issue of Cd contamination in rice grains, as it is very harmful to human health. Furthermore, crops grown in soil containing other metals such as Cs or Sr also pose a serious risk to humans [75]. Therefore, extensive information about the nature of different agriculturally important crops and their capacity to accumulate various elements through different routes, as well as complete information on their ionomes, is crucial for better human health. On the other hand, the deficiency of essential mineral nutrients causes dire symptoms, which can lead to complete mortality of plants under extreme conditions. However, when both essential and non-essential mineral elements are present at excess concentrations, they considerably reduce and inhibit plant growth [76] via biochemical, structural, and physiological changes [77]. In plants, heavy metal toxicity inhibits or alters many key biochemical or physiological traits such as photosynthesis, chlorosis, altered nutrient assimilation, low biomass accumulation, water balance, and senescence, eventually causing plant death [78]. In general, a plant can grow as long as the supply of a particular nutrient meets its needs. Hence, both mineral deficiency and excess have a major impact on food production and global food security. As ionomics can detect multiple elements, this technique is ideal for determining how changes in one element abundance influence others in plants. For example, numerous studies have demonstrated that increased Si concentrations can reduce Mn-mediated toxicity in a variety of crops [79,80]. Similarly, the impact of As on the nutrients present in rice plants can be determined using this method [81]. Based on previous findings, we have highlighted the importance of ionomics in modern agriculture (Figure 1).

## 3. Ionomics and Plant Biotic Stress

Plants are invaded by a number of pathogens, including fungi, oomycetes, bacteria, viruses, and insects. Plants use a complex multi-tier defense system to combat pathogens, and this is often triggered by ions such as Ca^2+^. Owing to the advent of ionomics, it is currently well recognized that mineral nutrients play an important role during plant-pathogen interactions. The minerals needed for pathogen survival are nearly identical to those required for plant survival [82]. Hence, the identification of new mediators of nutritional immunity in plants would require a thorough understanding of the dynamics and functional properties of mineral nutrients or the ionome during host–pathogen interactions. Generally, nutrients (micro and macro) play myriad roles in plants and are strictly regulated because many are toxic at high concentrations [83]. They boost structural strength and modulate various metabolic defense responses in host plants, thereby providing frontline defense against pathogens. The nutritional status of plants can be used to determine their susceptibility or resistance to pathogens [84]. Mineral nutrients are required to enhance growth and disease tolerance by activating key enzymes that generate prolific metabolites such as glucosinolates, lignin, callose, phytoalexins, and phenols. Previous studies have shown that hyper-accumulation of metal ions leads to effective disease resistance against *Pseudomonas syringae* in plants [85]. Interestingly, the concentration of minerals has a direct impact on the pathogen virulence or disease outcome. At low N concentrations, the impact of *P. syringae* disease severity was found to be low in tomato plants [86], whereas contrary results were observed for *Xanthomonas vesicatoria* infections [82]. Ca^2+^ ions are essential components of mineral nutrition that reduce disease severity or host susceptibility by inducing a large number of defense cascades, such as an increase in the rigidity of the cell membrane, because they are the key constituents of the cell wall (calcium galacturonates); hence, they increase cell stability [82]. In addition, Ca^2+^ significantly increases the activity of the antioxidant enzymes like superoxide dismutase (SOD), catalase (CAT), peroxidase (POD), and polyphenol oxidase (PPO) to scavenge reactive oxygen species (ROS) after pathogen infection [87]. This was demonstrated using plants that were Ca^2+^-deficient; many essential metabolites such as amino acids and sugars leaked out from the cytoplast region of the membrane to the apoplast, facilitating not only pathogen growth but also stimulation of disease progression [88]. Previous studies have revealed that hyper-accumulation of Zn ions is highly detrimental for bacterial pathogens, particularly *Xanthomonas* *fastidiosa*, because they play an important role in starch biosynthesis, protein structure, and protection of the plasma membrane from the effects of oxidative radicals [89]. In addition, Zn acts as a cofactor for countless enzymes that are mainly related to cell membrane integrity, hormone metabolism, and cell reproduction [83,90]. Similarly, Fe acts as a key source of competition between plants and pathogens during infection, to drive their cellular processes. Previous studies have shown that Fe deficiency in *A. thaliana* increases the expression of salicylic acid marker genes like *PR1* that might play a key role in reducing the disease incidence and development after infection with *Dickeya dadantii.* In contrast, when *A. thaliana* was supplemented with Fe, disease severity rose significantly because *D. dadantii* relies on its siderophore-mediated iron uptake system for systemic disease progression [91]. On the other hand, comparisons of leaf ionome between *Xylella fastidiosa* infected and non-infected olive trees show distinct differences, with Zn being the most discriminative ion between them and potentially serving as a biomarker for *X. fastidiosa* subsp. Pauca disease resistance [92]. In addition to their role in plant defense, minerals may have a significant impact on disease progression in plants because certain mineral nutrients help pathogen growth and survival. Nevertheless, they are obliquely involved, as they alter root exudates, determine microbial roles, and change the pH of the rhizosphere, and thus, have a huge impact on plant health and resilience [93]. The ionome has been used as an amalgamated phenotypic character to determine the relationship between pathogens and their host plants [94,95]. Previous ionomic studies have provided concrete evidence on the role of the ionome in disease resistance in lettuce against *Xanthomonas campestris* infections [96,97]. Attoma et al. [22] also showed that ionomics differentiates susceptibility and resistance traits in two olive cultivars that can further be used for identifying the target genes for olive disease management. Recent ionomics studies on grapes have revealed the intense restructuring of minerals in susceptible cultivars, indicating that minerals can play an essential protective role against pathogens [23]. On the other hand, pathogens employ a variety of defensive mechanisms to counteract the harmful effects of nutritional immunity. Previous ionomics studies have shown that the availability of nutrients has a significant impact on pathogen colonization in the corresponding host plants. For example, *Xanthomonas oryzae* pv. *oryzae* can force the host transcriptional machinery to activate and increase the expression levels of the Cu removal gene in rice, thereby facilitating its virulence [97]. Similarly, a bacterial pathogen of citrus greening disease hijacks host nutritional immunity by increasing the expression levels of small RNA linked with P starvation, thereby causing nutrient scarcity-like symptoms, a characteristic symptomatic feature in citrus [98]. In addition, sulfur (S) is an important constituent of plant defense compounds such as defensins, thionins, glucosinolates phytoalexins, glutathione, cysteine, and methionine, which are the major players of plant immune system. In addition, S based signaling molecules such as reactive sulfur species and hydrogen sulfide plays a key role in pathogen perception and signal transduction that are regulated by an array of plant defense hormones like SA, JA and ET [99]. For example, S based antioxidant like GSH plays multifaceted role in SA mediated defense response in plants. The production of one of the prime signaling molecules like ROS, hydrogen peroxide (H_2_O_2_) modifies the GSH/GSSG ratio and this change triggers SA-associated plant defense signaling by inducing the SA synthetic gene isochorismate synthase 1 (*ICS1*) [100]. Similarly, overexpression of *GSH1* in tobacco plants showed increased resistance to the bacterium Pst DC3000 [101]. Furthermore, S-based pathogenesis-related proteins such as defesins and thionins are important antifungal proteins and when overexpressed in model and crop transgenic plants confer effective disease resistance against a wide range of pathogens. In *A. thaliana* S based receptor, CRKs (Cys-rich receptor-like kinases) are induced after treatment with bacterial flagellin22 but its mutation promotes disease progression and host susceptibility to bacterial pathogens. However, constitutive expression of CRKs like *CRK28* increased disease resistance to bacterial pathogens in Arabidopsis [102]. Previous studies have shown that S based fertilizers provides diseases resistance against an array of pathogens [103,104]. Many studies have revealed the effect of K on disease progression or resistance in different crops. For example, when strawberries were grown under low K levels they were found to be more resistant to *Colletotrichum gloeosporioides* than those grown under high K levels, because low K levels leads to the production of ROS, as well as the activation of the jasmonic acid and ethylene pathways, all of which lead to disease resistance [105]. However, contrary results were found in *Cornus florida* L which was more resistant to fungal pathogen *Discula destructiva* under high K levels [106]. Several mineral-related genes have been identified in various crops, and all of them play important roles during host-pathogen interactions and can be used to develop next-generation disease-resistant crops for sustainable agriculture (Table 2). Based on these findings, ionomics can provide both theoretical and practical insights into plant disease, etiology, early and accurate diagnosis, and nutritional therapy. Therefore, more ionomics studies on different crops are required to understand various host-pathogen interactions, as they will provide a wealth of knowledge on their function and allow for the screening of resistant genotypes. Hence, combining ionomics with other omics will create new opportunities for identifying potent resistant genes to improve disease resistance in crops via genome editing.

## 4. Ionomics for Enhancement of Abiotic Stress Resilience in Crops

In agriculture, abiotic stresses have a detrimental effect on plant growth and development and cause changes in metabolism, especially in the uptake and translocation of essential mineral nutrients. Numerous inorganic elements have been reported to combat abiotic stress in various crops [131,132]. Although they are toxic at high concentrations, mineral nutrients and trace elements play a crucial role under the safe limit to mitigate the negative effects of abiotic stresses, namely, toxicity caused by drought, salinity, and heavy metals [90]. Ca^2+^, a multifaceted secondary signaling messenger, is involved in almost all abiotic stress-mediated signaling in plants. Exogenous application of Ca^2+^ to crops induces effective tolerance to an array of abiotic stresses [133]. Pankovic [134] highlighted that N (7.5 mM) in Helianthus plants reduces the deleterious impact of Cd on photosynthesis by elevating the activity of the Rubisco enzyme and increasing the accumulation of defense protein. Similarly, P is instrumental in decreasing the metal-induced noxiousness in crop plants, as it lowers metal concentrations or limits metal movement via the formation of metal-phosphate complexes [135]. The addition of 60 mg/kg K in *T. aestivum*, *L* significantly reduced the toxic effects caused by Cd by improving the concentration of non-enzymatic antioxidants (AsA and GSH). In another study, K supplementation alleviates Cd toxic effects in *Gladiolus grandiflora L.* by increasing the activity of antioxidant enzymes and proline, phenols, and flavonides [99]. Similarly, other mineral nutrients, including S, Mg, Zn, Fe, Se, and Si, have also been shown to lower heavy metal toxicity by boosting antioxidant activity, proline content, photosynthetic rate, and competing with heavy metal uptake, so minimizing their effect on cellular processes [90]. Under salt stress, GSH induced the ethylene level that ethylene plays a major role in stabilizing photosynthesis by maintaining the ROS accumulation, ion homeostasis, and mineral homeostasis and by elevating the antioxidant defense mechanism [136]. Similarly, S-induced GSH synthesis reduced ROS enhanced photosynthetic efficiency and growth in barley plants during salinity stress [137]. In maize plants, S based fertilizers improves drought tolerance by increasing not only the production of antioxidants but also increased rate of photosynthesis, stomatal conductance, and transpiration rate [138]. On the other hand, K plays a key role in drought tolerance in plants. For example, when sunflowers were grown under high K levels and drought conditions, stomatal closure is preceded from the guard cells via ethylene pathway and a higher photosynthetic rate was observed than under low K levels. In line with this, the ionomic profiling of guard cell reveals that B and Zn play a key role in stomata opening while Mn and Cd do in its closure, providing huge opportunities in understanding the significance of ions in the function of guard cells [139]. Previous studies have revealed that supplementation of N in wheat mitigates drought stress through the activation of an antioxidative defense system and higher photosynthetic activity [140]. Similarly, application of N on foliage and soil improved the growth and salinity tolerance of cotton [141]. In another study, application of P was found to combat salinity stress in wheat [142]. The effect of element uptake on different physiological components has been precisely evaluated in several studies to provide abiotic stress tolerance. Abiotic stress, such as salinity and/or heat, has previously been proven to cause alterations in nutrient intake. Generally, salinity stress increases Na^+^ uptake which has an antagonistic effect on the absorption of other ions K^+^, Ca^2+^ Mg^+2^, and NO^3−^, respectively. Similarly, an ionomic study in two recombinant inbred lines (RIL-66 low tolerant and RIL-76, high tolerant) of tomato illustrated a distinct profile between them with K^+^ and Mg^2+^ comparatively higher in RIL-76 than RIL-66 during salinity and heat combination. The important finding of this study was that K^+^ and Mg^2+^ can be used as heat and salt tolerant ionomic markers in tomato. However, no changes in N content were found between these the tolerant and sensitive RILs [143]. On the other hand, ionomic profiling of olive plants during drought stress shows how salicylic acid increases macro and micronutrients uptake, namely P, Fe, Mn, and Zn that mitigates the effect of drought stress by improving plant water relations, stomatal regulation, cell membrane stability, osmolyte accumulation, water use efficiency, as well as photosynthesis [144]. Abbas et al. [145] performed ionomic and physiological analysis to study the physiological and ionomic variation in Cd tolerant and sensitive maize genotypes. It was found that Cd tolerant cultivar had considerably higher proline, phenolics, and antioxidant accumulation, as well as higher uptake and translocation of N, P, K, Ca^2+^, Mg, Zn, and Fe from rhizosphere and root cell sap than Cd sensitive cultivar. These nutrients play a vital role in improving various physiological, biochemical, and molecular responses in plants for mitigating the harmful effects of stresses. Lee et al. [146] confirmed that canola genotypes with higher S usage efficiency (SUE) could effectively withstand drought stress. Similarly, drought tolerance was also observed in wheat following increased K accumulation [147]. In a recent study, Jia et al. [130] performed ionomic and metabolomic analyses of *Malus halliana* during saline-alkali stress, which caused ion imbalance and Na^+^ toxicity. These studies provided concrete evidence on the role of Ca^2+^-mediated signaling for maintaining Na^+^/K^+^ homeostasis, as well as reducing stress-induced injury. They also highlighted the role of key genes, such as those of ATP-binding cassette (*ABC*) transporters B1 and C10 (*ABCB1* and *ABCC10*, respectively), and *NatA* in mitigating salinity stress in *M. halliana*. Recently, ionomics profiling was used to investigate F tolerance in tea plants and it was found that F accumulation increases the production of Na^+^, Fe, Mn, and Mo in tea leaves that may provide the main positive charge to neutralize the negative charge from F thereby re-establishing ionic homeostasis [148]. On the other hand, F toxicity has been shown to reduce rice growth and yield by altering grain production [149]. Banerjee et al. [149] investigated the role of silicon nanoparticles (SiNPs) in ameliorating F toxicity in rice. They found that molecular priming with SiNPs effectively ameliorated injuries and improved yield by reducing F accumulation and increasing the content of non-enzymatic antioxidants such as glutathione, flavonoids, anthocyanins, and phenols in rice grains. Si priming also increases the levels of essential co-factors, namely, Fe, Zn, and Cu, which further enhance the activity of antioxidant enzymes such as catalase, superoxide dismutase, ascorbate peroxidase, and guaiacol peroxidase, thereby inducing F tolerance. Similarly, Si plays an important role in mitigating salinity stress in crops by augmenting the accumulation and activity of various physiological and biochemical defense components that further improve stress tolerance [150]. These results provide the concrete evidence that ions not only have nutritional benefits in plants but also provide stress resilience. Previous ionomics studies on various crops during abiotic stresses resulted in the discovery of a number of stress-resistant genes that could be used to generate elite stress-tolerant plants (Table 1).

## 5. Quantitative Trait Loci (QTLs) and Genes Regulating Ionomic Traits

The identification of genes/QTLs linked with the trait of interest is generally performed by QTL mapping and genome-wide association study (GWAS) of the crop species [151,152]. Ionomics evaluation of either a bi-parental mapping population or a diverse set of genotypes with molecular marker data enables efficient identification of the genomic loci governing a trait. The QTL mapping approach mostly uses a bi-parental mapping population [153,154]. In such a population, the profile of segregating molecular markers can correlate with any trait having variation among the parental lines. This approach can be used efficiently to identify loci for uptake and transport of different elements. The QTL mapping approach does not need any prior information about candidate genes and molecular pathways. Many QTL mapping studies have been carried out for the discovery of loci/genes associated with numerous ionomic traits (Table 3). For instance, QTLs for P accumulation [155], Cs accumulation [156] N uptake [157], and Al tolerance [158] have been identified in *A. thaliana*. Genetic mapping has also been used to identify the QTLs in rice, both in the leaves and grains, associated with 17 essential elements; 36 and 41 QTLs are associated with these elements in the leaves and grains, respectively. Buescher et al. [159] used high-throughput ICP-MS to analyze the amounts of various elements in 12 *Arabidopsis* accessions along with three recombinant inbred line (RIL) populations, which were developed under diverse environmental conditions. They observed significant differences among the accessions for most of the elements analyzed and found more than 100 QTLs for elemental accumulation in the RIL populations. It has been demonstrated that variations in the plant growth environment have a significant effect on the correlations among different elements and the QTLs controlling the ionome. QTL mapping studies for various ionomic traits have been performed for several crops such as *T. aestivum* [160], *B. napus* [161], and *Z. mays* [162]. Identification of the loci governing a trait has enormous importance in breeding applications, particularly in marker-assisted breeding. However, precise identification of the regulatory gene at the known locus using integrated omics tools is a prerequisite to understanding the underlying molecular mechanism [132,163]. For instance, once a QTL is defined, genomic data can be helpful to identify genes with the QTL region. However, most QTLs span thousands of bases and comprise hundreds of genes, which makes it challenging to determine the gene of interest [164]. Therefore, prior information about the gene functions in other plant species, or gene expression information, will be helpful to reduce the list to more favorable candidates. Earlier, a combination of QTL mapping and transcriptome profiling was found to be efficient in recognizing candidate genes [165,166,167]. Similarly, QTL mapping, genomic information, and gene expression data have been used by Induri et al. [168] to identify candidate genes in 16 QTLs linked to Cd accumulation in *Populus* spp.

The main shortcoming of QTL mapping is its approach of utilizing the variance between two or limited genotypes (i.e., bi-parental or multi-parent crosses). However, in a natural population, allelic diversity is very high; GWAS is an approach to identify significant genotype-to-phenotype links in order to manipulate such allelic diversity [175,176,177]. GWAS has been proven to be an important approach for isolating QTLs or single genes responsible for imparting phenotypic variation in complex quantitative traits such as yield, quality, and biotic and abiotic stress tolerance [178]. Numerous natural alleles that regulate ionomic traits have been identified using the GWAS approach. An extensive GWAS was performed for *A. thaliana*, and 96–192 accessions involving 107 traits were analyzed in addition to the leaf concentrations of 18 elements [179]. Single nucleotide polymorphisms (SNPs) linked with Na in leaves in the genomic region encompassing *HKT1;1*, a gene encoding a Na transporter, were identified. Nevertheless, owing to the insufficient number of samples (*n* = 93) used in the study, significant SNPs were not identified for other elements. Therefore, in another study, a larger set of 349 accessions was used to describe a substantial portion of the species-wide diversity. The accession sets were genotyped with 250,000 SNPs and used for GWAS, which recognized four ionomic loci, including *HKT1;1*, a locus having high potential for ensuring salinity tolerance [111]. Chao et al. [121] combined GWAS with transgenic complementation and linkage mapping and showed that natural variation for Cd accumulation in *A. thaliana* leaves is directed by allelic diversity in the coding region of *HMA3*. They also observed that in 349 accessions, a difference at the *HMA3* locus accounted for 30% of the overall variation in leaf Cd levels. Furthermore, *HMA4* has been reported to be an essential protein for Cd accumulation and tolerance in *A. thaliana* [180] and *A. halleri* [181]. In addition, GWAS has also been used to investigate the uptake of other elements such as As [124], S [124], Mo [179], and Na^+^ [111].

**Figure 2:**
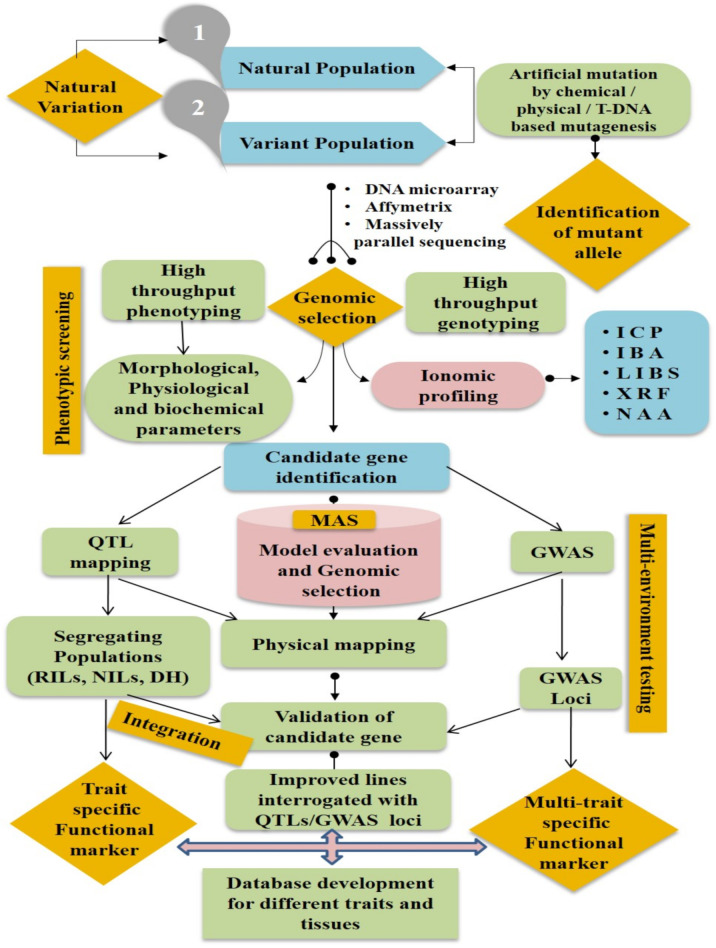
Quantitative trait locus (QTL) mapping and genome-wide association study (GWAS) for the identification of multi-trait specific biomarkers based on ionomic profiling and database development. RIL: recombinant inbred line; NIL: near isogenic line; DH: doubled haploid; MAS: marker-assisted selection; ICP: inductively coupled plasma; IBA: ion beam analysis; LIBS: laser-induced breakdown spectroscopy; XRF: X-ray fluorescence spectroscopy; NAA: neutron activation analysis.

In a recent investigation, Zhang et al. [182] performed GWAS of the concentrations of 13 elements in concert with a co-expression network. They identified 36 major QTLs related with 5 elemental concentrations that explained the phenotypic variation (PVE) from 18.35% to 27.56%. Twenty-four QTLs were identified for B, six for Na^+^, three for S, two for Cu, and one for Zn. Furthermore, 110 non-redundant candidate genes were discovered, and one of them (arahy.KQD4NT) was a metal transporter. Descalsota-Empleo et al. [183] carried out QTL mapping for 4 agronomic traits and 13 grain elements in 2 doubled-haploid (DH) populations, using a 6 K SNP chip. Further, 25 epistatic interactions were uncovered for 2 agronomic traits and 7 mineral elements. Some polished rice DH lines with high Fe and Zn contents were identified, and they could be used as donors for generating rice cultivars having high Zn content and in marker-assisted selection. Multi-elemental traits are affected by both genetic and environmental factors, and thus, should be correlated [184]. A total of 191 SNPs were identified by targeting high-yield and low-metal-toxicity genes in rice [185]. This offered new insights into the genetic basis of ionomic and agronomic variations in rice, as well as a foundation for marker production in crop breeding and further research into reducing heavy metal toxicity and improving crop yields. Therefore, a combination of multivariate and univariate QTL tools will provide additional knowledge that will aid in better understanding of the covariation of elements in the ionome and help uncover more genes that regulate elemental accumulation in plants (Figure 2).

## 6. Tools and Technological Advancements for Ionomics Studies

Initially, elemental profiling and distribution analysis were primarily used in environmental science and ecological studies. Ionomics has grown in popularity in the last decade as a tool for studying the metabolism and homeostasis of diverse ions or minerals in a variety of species, including plants and animals. However, this high-throughput ionomics tool requires sophisticated analytical instrumentation for quantitative and real-time measurement of elements in diverse tissue samples. Generally, techniques used in ionomics fall into two groups, which are mainly based on the atom electronic properties (emission, absorption, and fluorescence spectroscopy) or nuclear properties (radioactivity or atomic number) [14]. High-throughput analytical techniques such as liquid chromatography coupled to photodiode array/mass spectrometry (LC-PDA/MS), plasma optical emission spectroscopy (ICP-OES), liquid chromatography coupled to mass spectrometry (LC-MS), X-ray fluorescence (XRF), capillary electrophoresis coupled to mass spectrometry (CE-MS), gas chromatography coupled to mass spectrometry (GC-MS), nuclear magnetic resonance (NMR) spectroscopy, and Fourier transform-ion cyclotron resonance mass spectrometry (FT-ICR/MS) are sufficient and suitable for application in ionomics studies of plants [14]. Currently, ionomics relies on two high-throughput analytical techniques, namely ICP-MS and ICP-AES, owing mainly to their multiple elemental profiling with high accuracy. ICP-MS has been the technique most suitable for multi-elemental trace analysis of environmental samples, in the last few decades. This technique has several advantages over other techniques, such as the ability to detect very low concentrations of non-metallic elements, and it is regularly used for the detection of up to 18 elements in ionomics studies [186]. The detection limit of the ICP-MS is as low as parts per trillion (PPT), which facilitates the detection of trace elements in a minimal amount of test material. The advantage of ICP-MS over atomic absorption spectroscopy (AAS) is that it can quantify multiple elements simultaneously. Heavy metal pollution in soil and water is mostly studied by ICP-MS [187]. The applicability of ICP-MS to study heavy metal stress has been demonstrated in several studies. For example, Ardini et al. [188] used ICP-MS to determine the ionome in wild and mutant *Nicotiana langsdorffii* during heavy metal and drought stress. Based on their findings they have determined 29 major and trace elements from various parts of the plants with high accuracy and precision. Recently, this high throughput tool, along with laser capture microdissection (LCM), was used for multi-element analysis of nanogram-sized biological samples [189]. Data obtained using ICP-MS can be used to identify the loci that govern the uptake and transport of elements [190]. Cost-effectiveness and accuracy of quantification make ICP-MS a choice platform for geneticists, ecologists, and plant breeders. On the other hand, ICP-AES is generally used in plant ionomics analysis predominantly for the detection of Na^+^, K, Ca^2+^, Mg, P, Zn, Ba, Fe, Al, and Mn, which are present at higher levels in plants [191]. Neutron activation analysis (NAA) is an appropriate method for elemental profiling in environmental and agricultural investigations [192]. In NAA, the nucleus of a sample (target) is bombarded with a beam of neutrons, leading to the formation of radioactive isotopes. As the radioactive decay life of each element is known, the elemental composition and concentration in each sample can be identified using this method. In addition, this method is highly sensitive for ion detection; therefore, it can be used for the detection of elements that are present in low quantities [193]. NAA has been proven to be an appropriate technique for elemental profiling because it is fast and reliable for small sample analysis. Previous studies have shown the application of NAA for determining various elements such as (Al As, Br, Ca^2+^, Cd, Ce, Co, Cr, Cs, Cu, Fe, K, La, Na^+^, Rb, Sc, Sm, Sr, Zn, Lu, and Th) from different samples [194,195]. This technique has been proven to be an appropriate technique in ionomics studies where small samples of large numbers have to be analyzed. The general advantages of this method are that it can detect 60–70 elements with very good accuracy, it has a very large dynamic range, high selectivity and sensitivity, and it works with a single standard; however, it is the most time-consuming analytic technique [196]. However, expansion of other nonnuclear techniques like XRF and ICP-MS has led the decline of NAA method usage. Nonetheless, NAA is still useful in several applications, such as the analysis of various solid materials that are difficult to dissolve and the examination of samples to discover trace elements with low quantities [195]. X-Ray Fluorescence (XRF) is advantageous in environmental analysis. XRF spectroscopy is a multi-element technique, non-destructive, appropriate for several elements (Z > 18) with broad dynamic range, and economically practical. It can identify elements and determine concentrations in both liquid and solid samples [197]. X-rays (an external energy source) are used to illuminate a sample; in response, the sample emits X-ray photons of specific wavelengths that are used to determine the elemental composition. XRF has been used to identify various elements in *A. thaliana* [198]. Recent advances in XRF technology have made it more convenient to use in the field. Portable XRF (pXRF) is capable of quantifying a broad range of elements, including light elements such as Mg, Al, and Si [199]. An element such as Si, which is known to provide benefits against several abiotic stresses, can be efficiently quantified with pXRF [200,201]. XRF spectroscopy is frequently used for soil analysis and can also be utilized in plant analysis. Generally, elements such as Al (Z = 13), K (Z = 19), Ca^2+^ (Z = 20), Al (Z = 13), Ti (Z = 22), V (Z = 23), Mn (Z = 25), Cu (Z = 29), Zn (Z = 30), and Sr (Z = 38) can be determined using this method; in some cases, S (Z = 16), Cl (Z = 17), and Pb (Z = 82), as well as rarely, P (Z = 15), As (Z = 33), and Co (Z = 27), can be determined depending on the concentrations in the samples. Owing to advancements in ionomics tools, synchrotron-based X-ray fluorescence microscopy (SXRF) has become one of the most important elemental profiling analysis methods because it provides hidden information about ion homeostasis, such as how ions are taken up, transported, and processed in different cellular compartments [202]. This flexible technique also provides information about transporters and their effects on the element’s bioavailability. It also employs a multi-elemental detection system that collects data on several metals simultaneously without affecting the sample’s original state; hence, it can be used for multi-element analysis [203]. This high throughput tool has been used in various elemental profiling studies including the examination of Zn in mycorrhizae-infected tomato roots [204]; Se in the roots of onion [205], and Fe, Zn, Mn, and Cu distribution in rice grains [206,207]. Further, high throughput analytical techniques used in elemental profiling in different plants is shown in Table 4.

**Figure 3:**
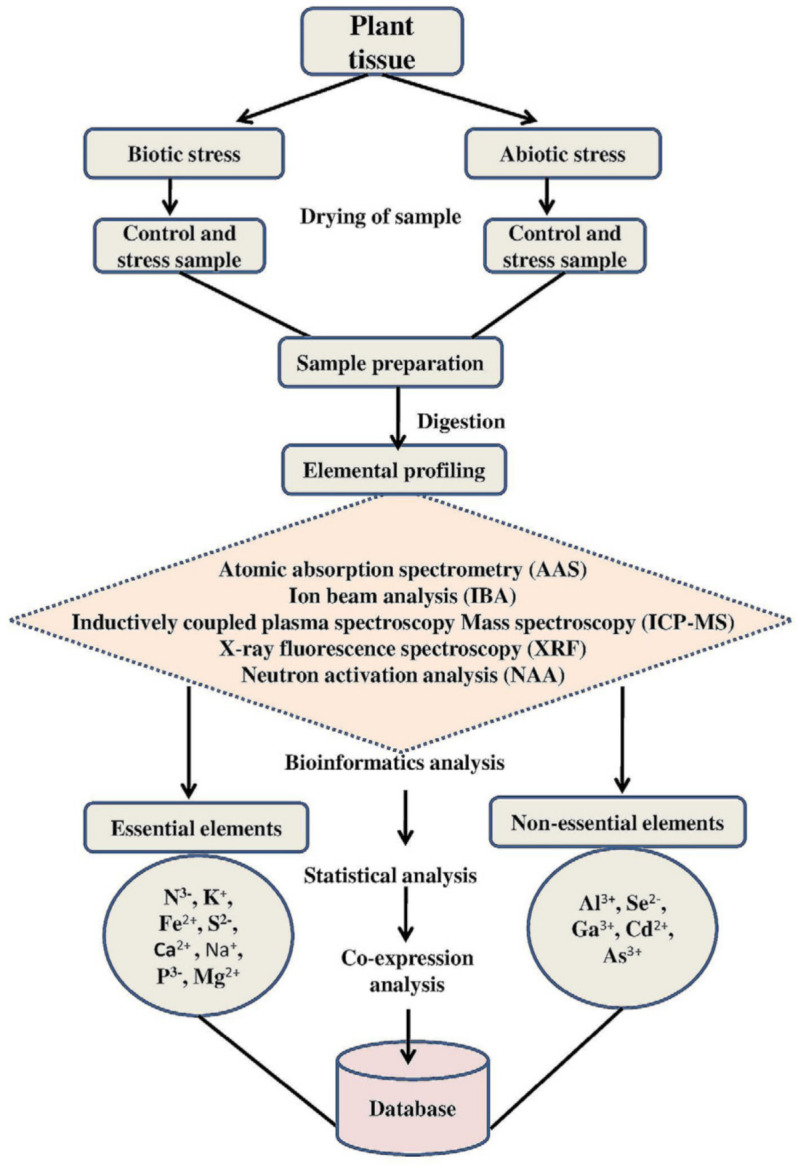
Description of ionomics workflow in plants under control and stress conditions. Sample preparation and elemental profiling using various analytical instruments is emphasized. The role of bioinformatics and other statistical methods to interpret ionomics data that can be stored in a database for future research is also highlighted.

Dragut et al. [219] developed an ionomics Atlas connecting the leaf ionome of an *A. thaliana* population, with respect to their landscape distribution. This ionomics Atlas consists of elemental data, the HapMap collection of 348 accessions of associated genetic loci, and properties of the habitat, such as geo-location and climate data (pressure, temperature, soil properties, humidity, and precipitation), where the accessions were collected, and can be accessed via iHUB. The ionomics Atlas helps researchers investigate the association of ionomics, genomic, and environmental data using the Google Maps application. Such tools enable the exploration of the possible role of natural ionomic variation in the landscape. In addition to data on *A. thaliana*, the iHUB contains ionomics data of economically important crops such as maize, rice, and soybean [219,220], and the eukaryotic model organism yeast [221]. Further, the iHUB tool can be used to study gene function and predict potential mutants for further analysis. As the amount of data produced by ionomics studies grows exponentially, visual interfaces and management systems for high-throughput ionomics data collection, storage, retrieval, and validation, and bioinformatics tools for data analysis, are becoming increasingly important. Furthermore, new ionome-based classification and prediction models need to be developed using machine learning algorithms and artificial neural networks (ANNs) to provide vital indicators for the early detection of stresses in sustainable agriculture. The procedure for analyzing and processing ionome data from various plant tissues under different environmental conditions using high-throughput platforms is shown in Figure 3.

## 7. Transporters and Channels Involved in Elemental Uptake and Transport

Plant nutrient contents are controlled by the balanced activities of membrane transporters that mediate nutrient uptake and distribution in order to maintain relative compositional homeostasis [222]. Ion accumulation is an intricate process that influences almost every stage of plant growth, as well as plant development and survival. The most important function of plant roots is sensing and absorption of nutrients, as well as essential mineral molecules, from the soil in order to sustain the growth and development of the plant. Many transporters, belonging to different transporter groups, mediate mineral uptake [223]. Herein, we discuss some of the important ion transporters and their regulation during various stress conditions.

Plants essentially require various metal ions such as Fe, Co, Zn, Cu, Ni, and Mn for growth and metabolic processes. The transport and accumulation of these metal ions in plants must be strictly regulated as excess ions can be deleterious to plant health. The accumulated metals are transported by specific transporters to various locations of the plant cell [224]. The major transporter proteins that are responsible for this process are heavy metal ATPases (HMAs), the natural resistance-associated macrophage protein (Nramp) family, the cation diffusion facilitator (CDF) family, the zinc-iron permease (ZIP) family, and the multidrug and toxin efflux (MATE) protein family and ATP-binding cassette (ABC) transporters [41]. HMAs are essential metal-transporting proteins that use ATP to pump transition metals such as Cu, Cd, Zn, Co, and Pb across the membranes. The heavy metal ATPases have two subclasses: CPx-type and P1B-type. The CPx-type ATPases are accountable for the transport of Cd, Zn, Pb, and Cu through the cell membranes [225], whereas P1B-type ATPases are responsible for regulating homeostasis and tolerance to metal accumulation, in addition to metal transport [226]. These HMAs, when overexpressed in various organs of plants, cause an increase in the accumulation of metals [227]. In terms of phylogeny, HMAs belongs to two major classes: the first class is Cu/Ag, and the other is Zn/Co/Cd/Pb [226]. In the case of plants, there are 8 and 9 copies of HMA genes in *O. sativa* and *A. thaliana,* respectively. The *AtHMA1* member of the first group is a chloroplastic protein that detoxifies Zn [228]. The *AtHMA1* deletion mutant is characterized by an inability to resist high Zn concentrations. Another member of this group, *AtHMA3*, is embedded in vacuoles and aids in the removal of Zn and Cd [229,230]. Additionally, it is also known to cause variability in the root and shoot tissues of various *Arabidopsis* species that overexpress *AtHMA3* and hence accumulate higher levels of Cd [121]. The *AtHMA2* and *AtHMA4* genes are embedded in the cell membrane and play a vital role in Cd and Zn detoxification [231,232,233,234]. Unlike dicots, there are few studies on HMAs from monocots. In rice, nine HMAs (*OsHMA1*, *OsHMA2, OsHMA3, OsHMA4*, *OsHMA5, OsHMA6, OsHMA7, OsHMA8*, and *OsHMA9*) were identified based on the genome sequence analysis [229]. *OsHMA1* shares 71% identity with *AtHMA1* and shows differential expression in rice plants under Zn-deficient and Zn-rich conditions, implying that it may be involved in Zn detoxification [235]. Previous studies have shown that *OsHMA2* is localized to the plasma membrane and is involved in the transport of Cd and Zn [236]. Similarly, *OsHMA3* was found to control of root-to-shoot Cd translocation rates [237]. In rice, *OsHMA4-OsHMA9* belongs to Cu/Ag subgroup of HMAs. Out of them, *OsHMA4* functions to sequester Cu into root vacuoles, limiting Cu accumulation in the grains [43]. Similarly, rice under high Cu concentrations highly expresses *OsHMA5*, hence might play a key role in Cu detoxification [238]. In addition, based on genome sequence analysis, 11 HMAs in maize and sorghum have been identified and are grouped according to the Arabidopsis and rice HMAs [239].

The second major metal transport protein family is the Nramp family. This family is present in diverse organisms ranging from simple-celled organisms such as bacteria to complex eukaryotes such as plants, animals, and fungi. In plants, the Nramp family was first identified in rice, which has three of these genes, followed by *Arabidopsis*, which has six copies [225,240]. Plants that encode *NRAMP* (natural resistance-associated macrophage protein) genes have been classified into two sub-families. The first group encodes *At*Nramps 1 and 6, whereas the second encodes *AtNRAMPs 2–5* [240,241]. *Os*Nramps 1 and 3 of rice fall in the first group, whereas *OsNRAMP 2* falls in the second group of the *NRAMP* family. CDFs are intermembrane transporters found in all living organisms which serve a vital role in the homeostasis of divalent metal cations including Zn^2+^, Fe^2+^, Cd^2+^, Co^2+^, Ni^2+^, Mn^2+^, and possibly Cu^2+^ and Pb^2+^. They have a transmembrane domain (TMD) and a cytoplasmic C-terminal domain (CTD) [242]. Plant CDFs are commonly called MTPs (Metal Tolerance Proteins) and have primarily been characterized as Mn^2+^ transport proteins. CDFs are divided into three types based on their metal ion selectivity. (i) Mn-CDF with Mn^2+^ as the only substrate (ii) Fe/Zn CDF with the Fe^2+^ and Zn^2+^ as well as other metal ions as substrates (iii) Zn-CDF with Zn^2+^ and other metal ions as substrates but not Fe^2+^ or Mn^2+^ [243]. As their role is primarily focused on effluxing, these transporters are also known as “cation efflux transporters” [244].

On the other hand, ZIP is a well-known transporter family that was first identified in plants and is competent of transporting an array of cations, including Fe, Zn, Cd, and Mn [244]. They are present in a variety of cell organelles and play an important role in both Zn homeostasis and plant adaptation to low and high Zn soils [245]. In addition, ZIP transporters also play a key role in Zn biofortification of grains. Interestingly, ZIP family transporter genes have been identified in many crops but are mainly validated in Arabidopsis (*AtZIP1*, *AtZIP2*, *AtZIP3*, and *AtZIP4*) [240,246] and rice plants (*OsZIP1*, *OsZIP3*, *OsZIP4*, *OsZIP5*, *OsZIP7*, and *OsZIP8*) [247]. MATE membrane effluxers are a primitive gene family of secondary transporters present in all living organisms but with a particularly large presence in plants. They play a key role in plant resistance to Al toxicity by inducing citrate efflux [248]. They also play a role in nutrient homeostasis in plants, such as Fe^3+^ uptake as well as the transport of secondary metabolites and hormones. Various MATE transporter genes have been identified in both model and crop plants like 56 in Arabidopsis [249], 117 in *G. max* [250], 70 in *M. truncatula* [251], and 45 in *O. sativa* [249]. Previously it was found that MATE transporter genes were upregulated in rice plants during Cd stress, suggesting that they could be involved in Cd detoxification [252]. Since MATE transporters play important roles in plant physiological processes, they may be good candidates for plant breeding programs aimed at improving agricultural traits like aluminum resistance, iron nutrition, and secondary metabolite accumulation [248].

ABC transporters comprise large group proteins that are found in microorganisms, plants, and animals [253]. Approximately 120 ABC transporter members in *Arabidopsis* and rice have been identified; they are grouped into seven subfamilies [254]. They are involved in diverse functions such as the transport of various molecules, including ions, lipids, carbohydrates, phytohormones, and heavy metals, across the cell membrane as well as across various cell organelles [240]. The ABC transporters are known to regulate several physiological processes such as transpiration, stomatal conductance, respiration, and photosynthesis in plants [255]. In addition to transporting various molecules, these transporters are known to transport various metals such as Cd, and thus, play a defensive role against heavy metal stress [256]. In plants, Cd is extremely toxic and can lead to nutrient homeostasis disturbance, DNA and membrane damage, protein dysfunction, and generation of noxious reactive oxygen species (ROS), all of which can lead to a substantial reduction in crop growth and productivity [257]. Overexpression of *AtABCC1* improves Cd tolerance and accumulation in *Arabidopsis*, whereas its knockout makes plants more vulnerable to Cd stress [258]. It is also known to detoxify Al in plants [259]. Hence, using ionomics tools, the role of different ABC transporters in mitigating heavy metal stresses in economically important crops can be further investigated.

As metalloids are both beneficial and toxic to plants, their precise regulation of uptake and translocation is crucial. Most metalloids are transported by aquaporins (AQPs), among which nodulin 26-like intrinsic protein-III (NIP-III), which belongs to the AQP family, has been found to be associated with the transportation of metalloids such as Si, B, Ge, and As [165,260]. NIP-IIIs are passive channels that facilitate the transportation of metalloids from the soil to roots. However, another class of transporter proteins, the arsenite-antimonite (arsb) efflux family, is involved in metalloid transport from the root to shoot [261,262]. The arsb family, also known as the Lsi2 (low silicon 2) family, comprises active efflux transporter proteins. As soon as NIP-IIIs take up Si from the soil within root cells, Lsi2 transports Si outside the cortical cells toward the stele. Once Si reaches the stele, it is further transported to areal tissues via xylem flow. NIP-IIIs and Lsi2s have been found in several plant species such as barley [263], soybean [264], maize [265], and wheat [266]. Ionomics tools such as ICP-MS and pXRF significantly help in the identification of loci and genes that govern the metalloid transportation process in plants [261,264]. Metalloids such as Si and B are known to enhance tolerance against several abiotic stresses. In particular, Si imparts resilience to plant species under biotic and abiotic stress conditions [166]. Several efforts have been made toward genome-wide identification of AQPs and their subsequent characterization into sub-classes, including metalloid transporter NIP-IIIs [165,201]. These studies have extensively used transcriptomics and genomics resources, but minimal information about ionomics has been integrated. Hence, using ionomics, it can be determined whether these transporters will take up other metalloids from the soil; this will provide novel insights for crop improvement.

## 8. Ionomics and Integrated Omics Approaches

**Figure 4:**
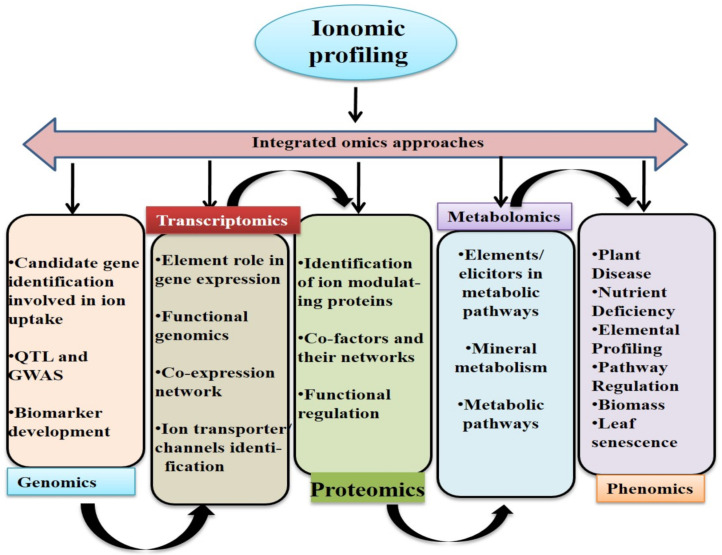
Ionomics and its integration with other omics approaches for identifying stress-resilient genes. Integration of multi-omics approaches provides the overall picture of the information flow from the upstream step of central dogma (genomics) to the last downstream level (metabolomics) that can define the phenotype. All omics are interdependent; hence, their integration will possibly allow the identification of potential genes and their regulatory networks, which can be used for developing smart crops for sustainable agriculture via genome editing or molecular breeding.

Multi-omics, along with other high-throughput tools, has revolutionized plant biology as it provides real-time readouts of hundreds of genes, proteins, metabolites, and ions at various developmental stages and under different environmental conditions. It possesses great potential to improve the nutritional status, as well as biotic and abiotic stress resilience, of plants. Although genomic tools can predict genes, genetic products such as proteins, metabolites, and ions can differ significantly due to transcriptional and post-transcriptional modifications. Hence, multi-omics will enable a better understanding of the metabolic networks, gene functions, and biochemical pathways, as well as their associations [214]. “Ionome”, “proteome”, “transcriptome”, and “metabolome” are four distinct terms that are interdependent and have overlapping boundaries [267]. These approaches produce a complex system of elements governed by the plant’s physiological and biochemical characteristics, which are in turn governed by the genome. In living systems, all processes are interconnected and equally important for the normal functioning of cells. In this regard, information derived from different omics approaches can help establish the inter-dependency of different cellular components, to draw a better picture of molecular phenomena [132,163]. To reconstruct complex networks that describe phenotypes in cells, ionomic profiling needs to be integrated with multiple omics data (Figure 4). A typical problem with ionomics data is a disparity in the correlated ions for their association with a biological function. To understand the complexity of a biochemical mechanism in cellular metabolism, as well as its regulation, a combination of transcriptomics data and metabolomic correlations is a suitable approach. Using publicly accessible data, further construction of gene-to-metabolite networks can be performed using in silico analysis of genes and metabolites. Multi-omics, in combination with high-throughput DNA sequencing, has allowed active investigations of networks that regulate biotic and abiotic stress responses, as well as studies on trait variation in different crop species [132,163,268].

A number of omics-based approaches, including ionomics, have been exploited in both model (Arabidopsis) and crop plants (wheat, rice, and tomato) to understand the underlying complexity of plant stress biology [269,270,271,272,273,274,275]. Ionomics and its integration with other omics have shown how organic N increases agricultural crop yield in an agroecosystem. This study highlights the importance of soil solarisation (SS) mediated increased growth response in *Brassica rapa* and it was found that SS boosted plant shoot biomass regardless of the type of fertilizer used. Further, using a multiomics approach, they identified soil organic N which is induced by soil solarization as one of the key components to increase crop yield in *B. rapa*. [276]. Another multi-omics approach shed light on combined yield-related traits and enhanced abiotic stress tolerance in tobacco [277]. Recently, ionomics and metabolomics studies revealed the role of P in wheat plants in improving drought stress tolerance [278]. Based on their findings, *T. aestivum* plants in the low phosphorus (LP) treatment were more drought tolerant than those in the conventional phosphorus (CP) treatment, which is likely due to the presence of a comprehensive mobilization of sugar metabolism, that regulates osmotic balance, as well as the accumulation of various organic acids, or the regulation of intracellular ion homeostasis. Interestingly, they also found the impact of different P supply on elements. *Viz.,* K, Ca^2+^, P, Si, Na^+^, Mn, Mg, and Zn which were differently regulated in presence of LP and CP treated plants during drought stress [278]. Subsequently, a variety of omics techniques has been successfully employed to better understand the genetic processes that regulate the plant ionome [279]. Guo et al. [280] provided an insight into the ionomics and transcriptomics of two cotton genotypes (sensitive and tolerant) under salinity stress. They found that the concentrations of Na^+^ in the roots, stems, and leaves increased significantly, whereas those of K, Cu, B, and Mo in the roots, as well as those of Mg and S in the leaves, decreased significantly under stress. Based on the ionomic profiling it was found that salt-sensitive cotton cultivar had more ions (Fe, Mn, Zn, S, K, Ca^2+^, and P) that were negatively related to Na^+^ than the salt-tolerant cultivar, respectively. Similarly, ionomic and metabolic profiling of maize seedlings carried out during neutral and alkaline salt stress has provided abundance information on the role of ions and metabolites [281]. Furthermore, integrated multi-omics analysis of barley root zones revealed two distinctive salt tolerant pathways based on the salt induced lignin and suberin impregnation in the root cells [282]. Grobikinsky et al. [283] studied the complicated molecular mechanisms of leaf senescence in model and non-model plant species using a multi-omics approach. Various efforts have been made to understand stress tolerance in tomato, soybean, brassica, barley, maize, wheat, and Arabidopsis using integrative omics-based approaches [132,163,193,279,284]. The impact of omics has provided an excellent platform to understand the pathways that regulate tolerance in grapevine upon exposure to both biotic and abiotic stress [285]. Ionomics and its integration with other omics approaches have been instrumental in elucidating the molecular complexity of plant stress biology and identifying target genes for developing smart crops for sustainable agriculture (Figure 4).

## 9. Conclusions

In the last decade, ionomics has grown in popularity as a high-throughput tool for studying the metabolism and homeostasis of ions or elements in a variety of species, including plants and animals. However, knowledge of the relationship between different ions or minerals and different stresses is limited. Being sessile, plants face numerous biotic and abiotic stresses, leading to negative effects on their growth, yield, and sustainable agricultural production. In this regard, many mineral nutrients, namely, N, P, K, S, Mg, Zn, Fe, Se, and Si, play a positive role in mitigating biotic and abiotic stresses. Traditionally, plant mineral analysis is performed manually and is expensive, time-consuming, less precise, and less reliable. However, the emergence of ionomics involving high-throughput elemental profiling of mineral nutrients and trace elements using various spectroscopic techniques has considerably increased the throughput and precision, as well as reduced the cost, of mineral analysis of crop plants. Owing to progressions in analytical tools and techniques, ionomics has emerged as a potentially favorable approach for sustainable crop improvement. Ionomics has successfully enabled the discovery of crucial genes, as well as the pathways, responsible for regulating mineral nutrient and trace element concentrations in the plant to present biotic and abiotic stress tolerance. These genes can be used to create smart crops with high nutritive value and greater resistance to environmental stresses through genome editing or molecular breeding. Ionomics has been proven to be a crucial method for the discovery of the loci that control ionomic variation naturally. Moreover, the current progress in “omics” approaches and strategies for their integration will lead to the creation of a novel genomic resource for developing economically important and stress-resistant crops for sustainable agriculture. In conclusion, the integration of omics with current breeding programs can lead to an evolution from genome-assisted breeding (GAB) to omics-assisted breeding (OAB) in future studies.

## Figures and Tables

**Table 1:** List of primary genes and their elemental targets that affect plant ionome in different plant species.

Species	Total Ionome Regulatory Genes	Primary Gene Names	Target Elements	Tissues	References
*A. thaliana*	136	*CNGC10*, *FRO2*, *MT1A*, *CPNIFS*, *LPCAT1*, *PHO1;H3*, *PCR2*, *BTSL2*, *FYVE1*, *CIPK23*, *ABCC1*, *CCC*, *NIP3;1*, *AtNRT1.5/ AtNPF7.3*, *AtMSA1*, *myb72*, *NAS4*, *PEN3*, *AtIRT3*, *AtAPR2*, *AtHMA5*, *AtAX1*, *MYB62*, *AtCITF1*, *BTSL1*, *PHT1;9*, *NIP6;1*, *AtNRAMP1*, *VIT1*, *SOS1/NHX7*, *ABH1*, *AtbZIP23*, *AtHMA4*, *AtHAC1*, *AtNRAMP3*, *AtMT4b*, *MOT1*, *FRU*, *ESB1*, *PHT1;5*, *PHO2*, *ZAT11*, *FPN1*, *PHT1;4*, *AtMTP11*, *AtMT4a*, *CNGC3*, *AtMTP1*, *BOR1*, *VIH2*, *TSC10a*, *NIP7*, *FRD3/MAN1*, *AtZIP1*, *myb10*, *CAX2*, *mir169a*, *AtCTL1*, *BTS*, *AtATPS1*, *bHLH34*, *PHO1*, *ZIFL2*, *PYE*, *AHA4*, *CAX3*, *SULTR3;1*, *bHLH38*, *bHLH39*, *AtMTP8*, *AtMTP3*, *MGT6*, *BOR2*, *GA1*, *AtHKT1;1*, *NIP5;1*, *HAK5*, *bHLH104*, *OPT3*, *IRT1*, *GSH1*, *YSL1*, *AtPHR1*, *AtHMA2*, *AtHMA3*, *CBL10*, *AtbZIP19*, *HMA1*, *NaKR1*, *ACR2*, *FPN2*, *MGT7*, *ZIF1*, *VIH1*, *CNGC2/DND1*, *APG5*, *AtSPL7*, *COPT5*, *SOS2*, *AtMfl1*, *PHT1;1*, *PCS1*, *CNGC1*, *YSL3*, *ILR3*, *AtTSB1*, *AtMYB36*, *COPT1*, *CPR5*, *AtNRAMP3*	K, Ca^2+^, Mg, Fe, Cd, Zn, As, Se, S, Zn, P, Mn, Co, K, Cd, Na, NO^3-^, Mo, Cu, B, Ni, Rb, Cs, Li, Sr	Root, shoot, leaf, seed	[29,31,32,33,34,35,36,37,38,39]
*O. sativa*	141	*OsMTP9*, *OsHKT1;5*, *AKT1*, *OsHORZ1*, *OsMGT1*, *OsHAC4*, *OsHMA4*, *OsNIP1;1*, *OsYSL2*, *OsYSL15*, *LSI1*, *OsMTP8.2*, *OsPHO1;2*, *OsPT2*, *OsRab6a*, *OsMTP8.1*, *OsMIT*, *OsNAS2*, *OsPHR2*, *OsHAK1*, *OsVIT1*, *OsYSL9*, *OsYSL16*, *OsHMA5*, *OsZIP3*, *OsABCC1*, *OsRMC*, *OsPCS1*, *OsZIP5*, *OsHRZ2*, *OsPHO2*, *OsPCS2*, *SPDT*, *OsHMA2*, *OsHKT2;1*, *TPKb*, *OsPHF1*, *OsHMA3*, *NRAMP5*, *OsMOT1;1*, *OsPRI1*, *OsNIP3;2*, *OsNIP3;3*, *OsATX1*, *OsVIT2*, *ZIFL12*, *OsMIR*, *OsbHLH133*, *OsBOR1*, *OsHRZ1*	Mn, Na^+^, K, Fe, Mg, Ás, Cu, Mn, Se, P, Zn, Cs, Cd, B	Leaf, shoot, root, seed	[40,41,42,43,44,45,46,47,48,49,50,51,52]
* T. aestivum *	267	*TaIPK1*, *Ta-PHR1*, *TaABCC13*, *HKT2;1*	Fe, Zn, P, Na^+^, Ca^2+^	Seed, root, shoot	[53,54,55,56,57]
* Z. mays *	152	*ZmHKT1*, *YS1*, *YS3*, *ZmHAK5*, *TLS1*	Na^+^, Fe, K, B	Leaf, root, shoot, anthers	[58,59,60,61,62,63]
*M. truncatula*	176	*MtMOT1.2*, *MtNramp1*, *MtMOT1.3*, *MtCOPT1*, *MtMTP2*, *MtZIP6*	Mo, Fe, Cu, Zn	Nodules	[64,65,66,67,68,69]

**Table 2:** List of genes identified in plants through high-throughput ionomics approaches.

Candidate Gene	Species Name	Role in Stress	Function	Related Elements	Reference
*FRD3* Ferric Reductase Defective3	*A. thaliana*	Biotic	Citrate transporter	High Mn^2+^ and Co^2+^	[107,108,109]
*HKT1;1* (High-affinity K+ transporter 1)	*A. thaliana*	Abiotic	Sodium transporter	High Na^+^	[110,111]
*APR2* (Adenylylphosphosulfate reductase)	*A. thaliana*	Sulfur Assimilation	50-Phosphosulfate reductase	High sulfate, S^2−^, and Se^2−^	[25,112]
*MOT1* (Molybdate transporter 1)	*A. thaliana*	Biotic and abiotic	Molybdenum transporter	Low Mo^2+^	[113,114]
*FPN2* (Ferroportin)	*A. thaliana*	Biotic and abiotic	Ferroportin metal efflux protein	High Co^2+^	[30]
*ESB1* (Enhanced suberin1)	*A. thaliana*	Biotic and abiotic	Dirigent domain-containing protein	Low Ca^2+^ and Mn^2+^; high Na^+^, S^2−^, K^+^, As^3+^, Se^2−^, and Mo^2+^	[115,116]
*NaKR1* (Sodium potassium root defective 1)	*A. thaliana*	Biotic and abiotic	Metal binding protein	High Na^+^, K^+^, and Rb^+^	[117]
*SGN1* (Schengen3)	*A. thaliana*	Biotic and abiotic	Kinase	High Mg^2+^	[117,118]
*CPR5* (Constitutive Expresser of Pathogenesis Related Genes 5)	*A. thaliana*	Biotic and abiotic	Constitutive expression of pathogen resistance	Low K^+^	[119]
*TSC10A* (Ketosphinganine reductase)	*A. thaliana*	Biotic and abiotic	3-Ketodihydrosphinganine reductase	Low Mg^2+^, Ca^2+^, Fe^2+^, and Mo^2+^; high Na^+^, K^+^, and Rb^+^	[120]
*HMA3* (Heavy Metal ATPase)	*A. thaliana*	Biotic and abiotic	Heavy metal ATPase	Low Cd^2+^	[121]
*ATPS1* (ATP sulfurylase 1)	*A. thaliana*	Biotic and abiotic	ATP sulfurylase	High sulfate	[122]
*SGN3* (Schengen3)	*Hordeum vulgare*	Biotic and abiotic	Receptor-like kinase	Low K^+^; high Mg^2+^	[123]
ATQ1/HAC1 (Arsenate reductase QTL1/High Arsenic Content 1)	*A. thaliana*	Biotic and abiotic	Arsenate reductase	High As^3+^	[124]
*MYB36* (MYB Domain protein 36)	*A. thaliana*	Biotic and abiotic	MYB domain transcription factor	Low Ca^2+^, Mn^2+^, and Fe^2+^; high Na^+^, Mg^2+^, and Zn^2+^	[28]
*GSL* (Glucosinolate)	*Arabidopsis halleri and Brassicaceae*	Biotic and abiotic	Increase in phloem of young leaves against *Myzuspersicae* protection in Arabidopsis	Zn^2+^, Cd^2+^	[125]
*AD* (Alcohol dehydrogenase)	*Cyamopsis tetragonoloba*	Biotic stress	Up-regulate Zn-binding AD and making pathogen resistant cultivar	Zn^2+^	[126]
*CA* (Carbonic anhydrase)	*A. thaliana*	Biotic stress	Act as salicylic acid binding protein	Zn^2+^	[127]
*MT* (Metallothionein-like protein)	*Fagus grandifolia*	Biotic stress	Resistant to fungal infection	Zn^2+^	[128]
*Znf* (Zn finger)	* T. aestivum *	Biotic stress	Key in the R-gene-specific resistance of plants to pathogens	Zn^2+^	[129]
*NHX8/ZTP1* (Na+/H+ transporter/Zinc transporter protein)	* Malus halliana *	Abiotic stress	Saline-alkali stress resistance	High Na^+^ and Fe^2+^	[130]
*AKT1*, *MRS2-4*, and *ZTP29*	* M. halliana *	Abiotic stress	Saline-alkali stress resistance	Low K^+^, Mg^2+^, and Zn^2+^	[130]
*ANT*, *ATP2A*, *CALM* and *SOS2*	* M. halliana *	Abiotic stress	Saline-alkali stress resistance	Ca^2+^ signal transduction	[130]

**Table 3:** List of different analytical platforms used in ionomics to study plant ionomes and identify loci/QTLs governing uptake and distribution of different elements in plant tissues.

Plant Species	Number of Genotypes	Plant Tissue Analyzed	Ionomic Tool Used for the Elemental Profiling	Elements Analyzed	Number of Most Significant Loci Associated with Ionomic Trait	Reference
Soybean	1653	Seeds	ICP-MS	K, P, Zn, Ca^2+^, Mg, Na^+^, S, Ni, Fe, Co, Al, Cu, Cd, Mo Se, Rb	573 unique SNPs	[169]
Rice	529	Seeds	ICP-MS	Ca^2+^, P, N, Na^+^, Mg, K, Zn, Cu, B, Cr, Mo, Cd, Mn, As, Pb, Co	72 loci	[170]
Rice	79	Seeds	Flow injection spectrophotometer, and ICPMS	P, Si, Fe, Zn, Cu, Mn, Ni, Pb, Mo, As, Co, Cd, Al, Se	36 QTLs	[171]
Common bean	84	Seeds	ICP-AES	Fe, S, Ca^2+^, Mg, Cu, Zn, Ni, Mo, Mn, B, Cd, Co,	21 QTLs	[172]
Monkey flower	168	Leaves	ICP-MS	K, P, Ca ^2+^ , Na ^+^ , S, Zn, Mg, Fe, Mn, Cu, Rb, B, Sr, Se, As, Cd, Ni, Li, Mo	7 QTL	[173]
Barley	336	Grains	ICP-MS	P, S, Si, Na^+^, Fe, Ba, Mn, Mg, Ca^2+^, Sr, Zn, Cu	15 SNP loci	[174]

**Table 4:** Shows the high-throughput analytical techniques commonly used for ionomics in plants.

Plant species	Ion	Medium	Tissue Used	Platform	Study	Reference
*A. thaliana*	B, Ca^2+^, Mg, Mn, Fe, Cu, Zn, P, Co, Mo, As, Cd	Pot assay	Leaf	ICP-MS	Fe and P homeostasis	[24]
Rice *(O. sativa)*	6 elements K, Ca^2+^, Mn, Zn, Cu, Fe	Lab/tissue culture condition	Seed	SXRF	Characterization of a new Zn plasma membrane transporter, OsZIP7	[208]
Apple *(M. halliana)*	Ca^2+^, Fe, Zn, Mg, Mn, Na^+^, K, Cu, Cl	Hydroponics	Seedlings	ICP-OES, Ion exchange chromatography, LC-MS	To study saline-alkali stress in *M. halliana* seedlings	[130]
Soybean *(G.max)*	B, Na^+^, Mg, Al, P, S, K, Ca^2+^, Mn, Fe, Co, Ni, Cu, Zn, As, Se, Rb, Mo, and Cd	Field conditions	Seed	ICP-MS and SoySNP50k chip data	Identified candidate SNPs controlling elemental accumulation as well as lines with extreme elemental accumulation phenotypes.	[169]
Soybean *(G. max)*	Se, Cu, Fe, Mn	Field conditions	Seed	ICP-MS	C/N and other elements	[209]
Tomato *(Solanum lycopersicum)*	Na^+^ and Cl	Sand culture	Root, stem and leaf	AAS	Role of Si in mitigating abiotic stress	[210]
Lotus *(Lotus japonicus)*	15 elements B, Cd, Ca^2+^, Cu, Cs, Fe, Pb, Mg, Mn, Mo, Ni, K, Na^+^, Sr and Zn	Hydroponics	Seeds	ICP-MS	To investigate the accumulation of 15 elements in shoots of mutants of *L. japonicus*	[211]
Barley *(H. vulgare)*	Na^+^, K, Ca^2+^, Mg, P, S, Cu, Fe, Mn, and Zn	Hydroponics and pot conditions	Germination/seedlings	ICP-OES	Salinity stress in barley	[212]
Breckland wormwood *(Artemisia campestris)*	21 elements Higher: K, Ca^2+^, Fe, Na^+^ As, Ba, Br, Ca, Ce, Co, Cr, Cs, Eu, Fe, Hf, K, La, Na, Rb, Sb, Sc, Sm, Sr, Yb, Zn	Field conditions	Leaf	NAA	Determining essential and toxic elements	[213]
Tobacco *(N. angsdorffii)*	29 elements Ba, Bi, Ca^2+^, Cd, Co, Cr, Cu, Eu, Fe, Ga, K, Li, Mg, Mn, Mo, Na^+^, P, Pb, Pt, Rb, S, Sb, Sn, Sr, Te, V, W, Y, and Zn	Agar medium	Root, stem and leaves	ICP-AES/MS	Ionomic profiling of *N. langsdorffii* wild-type and mutant genotypes exposed to abiotic stresses	[188]
Soybean *(Glycine soja* * (L.) Merr. and G. max) *	Br, Cl, and I	Soil	Seeds	ICP-MS	Determination of bromine, chlorine, and iodine in soybean	[214]
* A. halleri *	Zn, Cd	Pot conditions	Leaf	HPLC, ICP-AES	To investigate the effects of the heavy metals Zn and/or Cd on aphid in *A. halleri*	[125]
*A. thaliana*	Zn	Growth chamber	Leaf tissue	LC-MS/MS	Role in plant immunity	[127]
Wheat *(T. aestivum)*	Zn	Pot conditions	Seedlings	HPLC-MS	key in the R-gene-specific resistance of plants to pathogens	[129]
Soybean *(G. soja)*	Na^+^, K, Ca^2+^, and Mg	Field	Roots, shoots, leaves, seeds, and capsules	AAS	The K-Na ratio of seed, leaf, shoot, and capsule were all >1 in the wild	[215]
Lotus Extremophile *(Lotus creticus) and* glycophytic *(Lotus corniculatus and Lotus tenuis. L.)*	Ca^2+^, B, P, Mn, S, Zn, Mg, Fe, Cl, K, Na^+^ (elements) Proline, serine, sucrose, Glyceric acid, citric acid, succinic acid, erythronic acid (metabolites)	Pot and filed conditions	Complete shoots (pooling leaves, petioles, and stems)	Gas chromatography coupled to electron impact ionization-time of flight-mass spectrometry (GC/EI-TOF-MS)	Glyphocites adapted well under salinity	[216]
Soybean *(G. max)*	B, Na^+^, Mg, Al, P, S, K, Ca^2+^, Mn, Fe, Co, Ni, Cu, Zn, As, Se, Rb, Mo, and Cd	Filed conditions	Seeds	ICP-MS	Ionomic screening for identifying mutant soybean lines with altered elemental composition	[18]
Soybean *(G. max)*	B, Al, Mn, Fe, Co, Ni, Cu, Zn, Sr, Mo, and Ba,	Soil	Seeds	ICP-MS and NMR	Elemental and lipid profiling of transgenic (cp4-EPSPS gene) and wild type soybean seed generations	[217]
* Acacia catechu * , *Argemone mexicana*, *Aegle marmelos*, *Datura metel*, *Phyllanthus emblica*, *Sapindus emarginatus*, *Senna occidentalis*	13 elements Major elements found: K, Ca^2+^, Cl, S, P Lower elements: Cu, Zn, Fe, Mn, Se, Br, Rb, Sr	Field conditions	Leaves, bark, fruits	XRF	Phytomedicine	[218]
